# Danshensu alleviates bleomycin-induced pulmonary fibrosis by inhibiting lung fibroblast-to-myofibroblast transition via the MEK/ERK signaling pathway

**DOI:** 10.1080/21655979.2021.1944020

**Published:** 2021-06-30

**Authors:** Huaman Liu, Xinyue Zhang, Yumeng Shao, Xuehong Lin, Feng Dong, Xue Liu

**Affiliations:** a College of Traditional Chinese Medicine, Shandong University of Traditional Chinese Medicine, Jinan, China; b Department of General Medicine, The Affiliated Hospital of Shandong University of Traditional Chinese Medicine, Jinan, China; c First College of Clinical Medicine, Shandong University of Traditional Chinese Medicine, Jinan, China; d Department of Respiration, The Affiliated Hospital of Shandong University of Traditional Chinese Medicine, Jinan, China

**Keywords:** Pulmonary fibrosis, danshensu, fibroblast, myofibroblas, MEK; ERK

## Abstract

Pulmonary fibrosis (PF) is a chronic pulmonary interstitial disease, and its pathological process is closely related to fibroblast–myofibroblast differentiation. Danshensu (DSS) has been reported to exert an anti-fibrotic effect in heart and liver. However, it is unknown whether DSS has an equally anti-fibrotic effect on lungs. To evaluate the effect of DSS on PF and demonstrate its possible molecular mechanisms, we established an *in vitro* model on TGF-β1 (5 ng/mL)-stimulated NIH3T3 cells and *in vivo* model on bleomycin (BLM) (5 mg/kg)-induced PF mice. *In vitro*, our results revealed that 50 μM DSS effectively inhibited the fibroblast proliferation, migration and differentiation into myofibroblast. *In vivo*, our results showed that DSS (28 and 56 mg/kg) reduced damaged lung structures, infiltrated inflammatory cells and accumulated areas of collagen deposition. Moreover, we showed that DSS decreased the fibroblast-specific protein 1 (FSP-1) – and α-SMA-positive areas. Meanwhile, we indicated that DSS reduced the expression of TGF-β1, α-SMA and COL-I in the lung tissues of mice. To further explore the mechanism of DSS on alleviating PF, we detected the MEK/ERK signaling pathway. Our results showed that DSS reduced the phosphorylation of MEK1/2 and ERK1/2, indicating that DSS might inhibit the MEK/ERK signaling pathway. Taken together, these results demonstrated that DSS could suppress lung fibroblast proliferation, migration and differentiation to myofibroblasts, possibly through suppressing the MEK/ERK signaling pathway, which suggested that DSS might be a potential therapeutic drug for PF treatment.

## Grafical abstract

**Figure:**
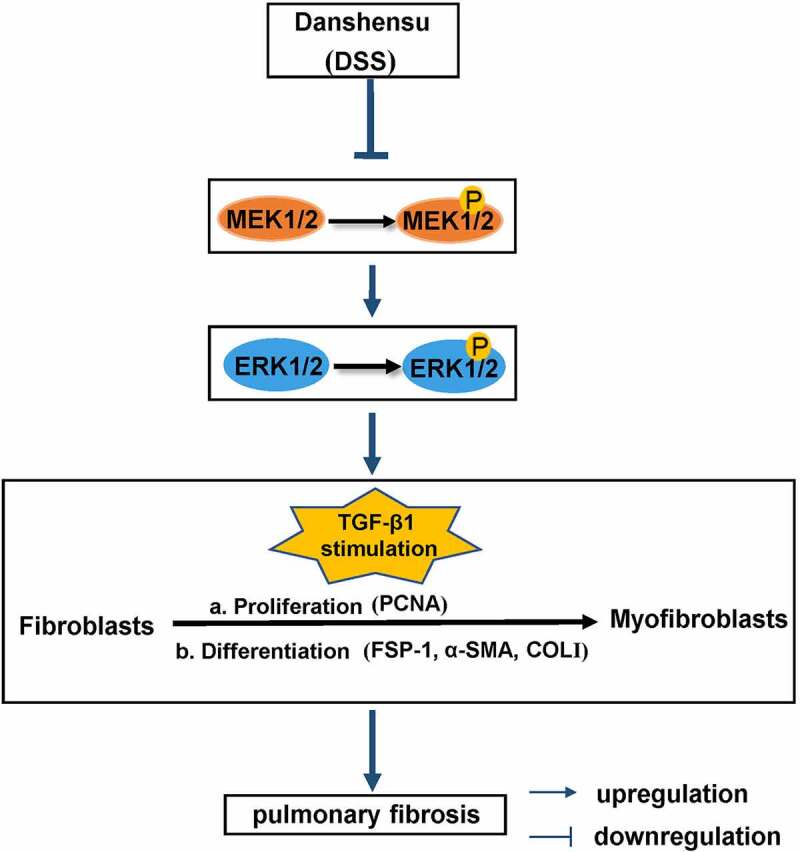

## Introduction

Pulmonary fibrosis (PF), as a chronic pulmonary interstitial disease, is characterized by aberrant fibroblast proliferation, excessive extracellular matrix (ECM) accumulation, inflammatory damage and tissue structure destruction, which ultimately leads to lung scarring formation, pulmonary insufficiency and respiratory failure [1,2]. PF has high mortality and poor prognosis with unknown causes [3,4]. Several explanations including oxidative stress [5], inflammatory response [6,7], fibroblast activation and abnormal deposition of ECM [8] have been involved in the pathogenesis of PF. Importantly, the progression of fibroblasts differentiating into myofibroblasts is likely the key to PF [4]. Under the stimulation of pathological factors such as TGF-β1^8^, fibroblasts can proliferate and differentiate into α-smooth muscle actin (α-SMA)-positive myofibroblasts in large numbers [9,10]. These myofibroblasts acquire an aggressive phenotype followed by the accumulation of type collagen , abnormal deposition of ECM and acceleration of PF remodeling [11,12]. Therefore, to explore new ways for alleviating PF is of great significance.

Danshensu (DSS), one of the main active ingredients in Danshen, has various pharmacological activities such as anti-apoptosis, anti-oxidation, and anti-inflammation [13]. Particularly, Luo et al. have found that Salvia miltiorrhiza Bunge (containing DSS) can relieve inflammation and airway remodeling in asthma model mice, and inhibit TGF-β1-induced epithelial-mesenchymal transition (EMT) and fibrosis in BEAS-2B and MRC-5 cells [14]. Moreover, several studies have shown that DSS can improve liver fibrosis and myocardial fibrosis by inhibiting oxidative stress response [15,16]. Hence, we hypothesized that DSS might attenuate PF via inhibiting fibroblast-myofibroblast differentiation.

To explore the role of DSS in PF, we first established an *in vitro* model on TGF-β1 (5 ng/mL)-stimulated NIH3T3 cells and determined the function of DSS on the fibroblast proliferation, migration and differentiation into myofibroblast. Furthermore, we performed an *in vivo* model on bleomycin (BLM) (5 mg/kg)-induced PF mice to evaluate the effect of DSS on lung structures and collagen deposition. Finally, western blot assay was used to detect the MEK/ERK signaling pathway.

## Materials and methods

### Cell culture

DSS (purity >98%; molecular weight 198.17), purchased from Meilunbio (Dalian, China), was dissolved in sterile distilled water as a 40-mM stock solution. NIH3T3 cells (Procell Life Science & Technology Co., Ltd., Wuhan, China) were cultured in Dulbecco’s modified eagle medium (DMEM, Gbico, USA) containing 10% fetal bovine serum (FBS) in a 37°C, 5% CO_2_ incubator. Then, NIH3T3 cells were treated in different ways as followed: part of NIH3T3 cells were treated with DSS at different concentrations (0, 25, 50, 100, 200, and 400 μM) for 24 h; part of NIH3T3 cells were pretreated with various concentrations of DSS (0, 25, 50, 100, 200, and 400 μM) for 2 h, followed by stimulation with or without TGF-β1 (5 ng/mL) for 24 h and another part of NIH3T3 cells were pretreated with DSS (50 μM) for 2 h, followed by the stimulation with or without TGF-β1 (5 ng/mL) for 24 h.

### Cell Counting Kit-8 (CCK-8) assay

The CCK-8 kit (KGA317, Keygenbio, Nanjing, China) was used to assess cell viability and cell proliferation. NIH3T3 cells were seeded in 96-well plates at a density of 5 × 10^3^ cells per well. After NIH3T3 cells were treated, the CCK-8 solution (10 μL) was added to each well and incubated at 37°C for 2 h. The optical density was read at a wavelength of 450 nm with amicroplate reader (800Ts, BIOTEK, USA).

### Wound healing assay

NIH3T3 cells were seeded in 6-well plates. When the cell density reached about 90%, the medium was replaced with serum-free medium and treated with 1 μg/mL mitomycin C (M0503, Sigma-Aldrich, USA) for 1 h. Then, a 200 μL pipette tip was used to scratch the cells. The wounded gaps were photographed at 0 h and 24 h under a microscope at a magnification of 100× (DP73, OLYMPUS, Japan) and the wound healing ratio of each group was calculated.

### Quantitative real-time PCR (qRT-PCR)

Total RNA was isolated from NIH3T3 cells by using Total RNA fast extraction kit (RP1202, BioTeke, China) following the manufacturer’s protocol. RNA concentration was determined using the UV spectrophotometer NANO 2000 (Thermo, USA). The RNAs were reverse transcribed into cDNAs, which was performed by using Super M-MLV Reverse Transcriptase (BioTeke) according to the manufacturer’ s instructions. Real-time PCR was performed in 20 μL reactions on cDNA with SYBR Green (EP1602, BioTeke) and Taq HS Perfect Mix (R300A, Takara, Japan) by using Exicycler^TM^ 96 Real-time Quantitative Thermal Block (BIONEER, Korea). The level of target mRNA was normalized to the level of GAPDH. Data were analyzed with a 2^−ΔΔCT^ method. Each gene analysis was performed in triplicate. Primer sequences of the targeted genes were listed as followed: Col1a1 forward: CGCCATCAAGGTCTACTGC, Col1a1 reverse: GAATCCATCGGTCATGCTCT; α-SMA forward: GACGCTGAAGTATCCGATAGAACACG, α-SMA reverse: CACCATCTCCAGAGTCCAGCACAAT; GAPDH forward: TGTTCCTACCCCCAATGTGTCCGTC, GAPDH reverse: CTGGTCCTCAGTGTAGCCCAAGATG.

### Immunofluorescence staining

NIH3T3 cells were first fixed with 4% formaldehyde for 15 min at room temperature (RT). After treated with 0.1% Triton X-100 (ST795, Beyotime, China) for 30 min at RT, NIH3T3 cells were blocked with goat serum (SL038, Solarbio, China) at RT for 15 min followed by an incubation with the primary antibody against α-SMA (1:200; AF1032, Affinity, China) overnight at 4°C. Next, the cells were incubated with Cy3-labeled goat anti-rabbit IgG (1:200, A0516, Affinity) for 1 h at RT. DAPI was used to counter-stain cell nuclei for 5 min at 37°C, and the cells were photographed under a microscope at a magnification of 400 × .

### Animals

C57BL/6 male mice aged 7–8 weeks were purchased from Liaoning Changsheng biotechnology co., Ltd (Liaoning, China). All mice were maintained under standard conditions at our animal facility (temperature of 22 ± 1°C, humidity of 45–55% and a 12-h light/dark cycle) with a specific pathogen-free environment, free access to food and water. All animal experiments were approved by the Animal Research Ethics Board of The Affiliated Hospital of Shandong University (Permission Number: 2019–58), in accord with the Guide for the Care and Use of Laboratory Animals.

### Establishment of PF induced by BLM in mice

The experiment of BLM-induced PF in mice was performed as previously reported [17]. Briefly, after a week of adaptive feeding, C57BL/6 mice were divided randomly into four groups: (1) the control group; (2) the BLM group; (3) BLM + DSS (28 mg/kg) group; (4) BLM + DSS (56 mg/kg) group. A single intratracheal instillation of BLM (5 mg/kg) was performed to induce PF in mice. Mice in the control group received an equal volume of saline. One day after BLM treatment, mice in the BLM + DSS group were intragastrically administration of DSS (28 mg/kg or 56 mg/kg) while mice in BLM group were intragastrically administrated with equal volume 0.9% sterilized salin. After 3 weeks, mice were sacrificed and lung tissues were collected and measured. The pulmonary index was calculated using the following formula: Pulmonary index = (Lung weight (mg)) ⁄ (Body weight (g)) ×100%.

### Hematoxylin-eosin (HE) and sirusred staining

The lung tissues were fixed with 4% paraformaldehyde, dehydrated in 70% (2 h), 80% (overnight), 90% (2 h), 100% I (1 h), 100% II (1 h) gradient alcohol, permeabilized in xylene for 30 min, embedded in paraffin, and cut into 5-μm slices for subsequent experiments. After deparaffinization and hydration, the slices were heated with the antigen retrieval solution for 10 min. Then, the slices were stained with hematoxylin and eosin (WLA051a, Wanleibio, China) and sirusred (S8060, Solarbio, China) respectively. Finally, the slices were mounted for observation under a microscope.

### Immunohistochemistry (IHC)

IHC was performed to evaluate the fibrotic phenotype of lung tissues. After deparaffinization and hydration, the slices were heated with the antigen retrieval solution for 10 min. Then, the slices were penetrated with PBS for 5 min followed with H_2_O_2_ treatment for about 15 min, and then blocked with goat serum (SL038, Solarbio) for 60 min at RT to eliminate the activity of endogenous peroxidase. The slices were incubated overnight at 4°C with antibodies against the following proteins: α-SMA (1:200, AF1032, Affinity) and fibroblast-specific protein 1 (FSP-1, 1: 200, A19109, Abclonal). The next day, the slices were incubated with HRP-labeled secondary antibody (1:500, #31460, ThermoFisher, USA) at 37°C for 60 min. After developed with DAB reagent (DA1010, Solarbio), the slices were then counterstained with hematoxylin (H8070, Solarbio). Finally, the slides were mounted and observed under a microscope.

### Western blot

Proteins were extracted by mixing RIPA lysate (R0010, Solarbio) and PMSF (P0100, Solarbio). The concentrations of proteins were assayed by the BCA kit (PC0020, Solarbio). According to different molecular weights, 5% concentrated gel and 8% separation gel concentrations were used in the SDS-PAGE. After transferred to a PVDF membrane, the proteins were blocked by prepared 5% (M/V) BSA (Biosharp, BS043, China) in TBST buffer for 1 h and incubated overnight at 4 °C with the following primary antibodies: α-SMA (1: 500), COL-I (1: 500, AF0134, Affinity), TGF-β1 (1: 1000, AF1027, Affinity), proliferating cell nuclear antigen (PCNA) (1: 500, A12427, Abclonal, China), p-MEK1/2 (1: 500, AP0209, Abclonal), MEK1/2 (1: 500, A4868, Abclonal), p-ERK1/2 (1: 500, AF1015, Affinity), ERK1/2 (1: 500, AF6240, Affinity) and GAPDH (1: 10000, 60004-1-Ig, Proteintech, China). Next, the membrane was incubated with goat anti-mouse IgG (1: 3000, SE131, Solarbio) or goat anti-rabbit IgG (1: 3000, SE134, Solarbio) secondary antibody for 40 min at 37 °C. At last, the specific protein bands were visualized with Western ECL Substrate (D1010, Solarbio).

### Statistical analysis

Data were expressed as mean ± standard deviation (SD). Statistical significance was assessed by one-way analysis of variance (ANOVA). P < 0.05 was considered statistically significant.

## Results

To explore the function and mechanism of DSS in PF, we established an *in vitro* model on TGF-β1 (5 ng/mL)-stimulated NIH3T3 cells and an *in vivo* model on bleomycin (BLM) (5 mg/kg)-induced PF mice. Functionally, we determined the fibroblast proliferation, migration and differentiation into myofibroblast *in vitro*. Moreover, we evaluated the effect of DSS on lung structures and collagen deposition *in vivo*. Mechanically, western blot assay was used to detect the MEK/ERK signaling pathway.

### Effects of DSS on the migration and proliferation of NIH3T3 cells

**Figure 1.**
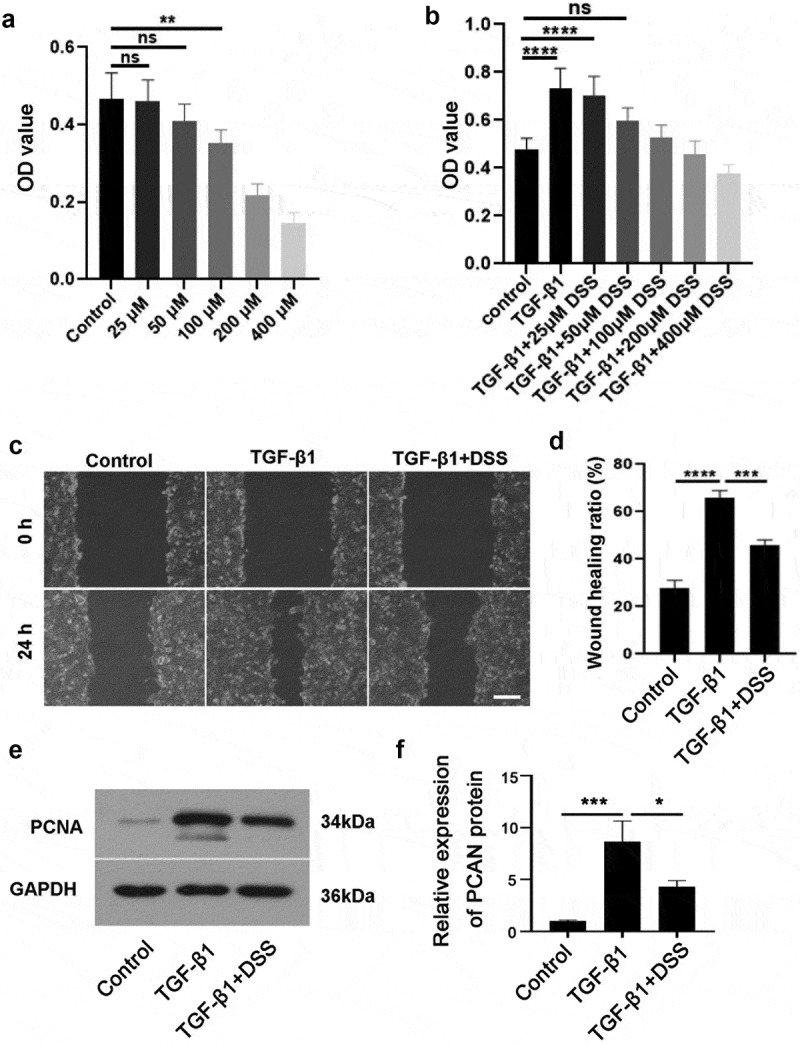
DSS repressed the proliferation and migration of NIH3T3 cells induced by TGF-β1. NIH3T3 cells were treated with TGF-β1 (5 ng/mL) in the absence or presence of DSS for 24 h. (a) The effect of DSS (25, 50, 100, 200 and 400 μM) on the cell viability was detected by CCK-8 assay. (b) The effect of DSS (25, 50, 100, 200 and 400 μM) on the cell viability induced by TGF-β1 was detected by CCK-8 assay. (c) Effects of DSS (50 μM) on the cell migration were detected by wound healing analysis. Scale bar: 200 μm. (d) The calculation of wound healing ratio. (e, f) The expression of PCNA was detected by western blot assay. GAPDH was conducted as a loading control. One-way ANOVA, *p < 0.05, **p < 0.01, ***p < 0.001, ****p < 0.0001, ns: non-significant

CCK-8 assay was used to select the appropriate concentration of DSS for subsequent experiments. Our results showed that compared with the control cells, there was no significant difference in the effects of 25 μM and 50 μM DSS on cell viability (Figure 1a). Besides, 50 μM DSS had a better effect on reducing cell proliferation induced by TGF-β1 (Figure 1b). Hence, 50 μM DSS was selected for subsequent experiments. To further verify the role of DSS in the migration and proliferation of TGF-β1-induced NIH3T3 cells, we performed wound healing assay and detected the expression of proliferation-related protein PCNA. Our results showed that DSS significantly reduced the migration of NIH3T3 cells induced by TGF-β1 (Figure 1c and Figure 1d) (p < 0.01). Western blot result indicated that TGF-β1 promoted PCNA expression, while DSS reversed this result (Figure 1e and Figure 1f). Collectively, these results revealed that DSS suppressed the migration and proliferation in TGF-β1-induced NIH3T3 cells.

### Effects of DSS on fibroblast-myofibroblast differentiation and the MEK/ERK signaling pathway in TGF-β1-induced NIH3T3 cells

**Figure 2.**
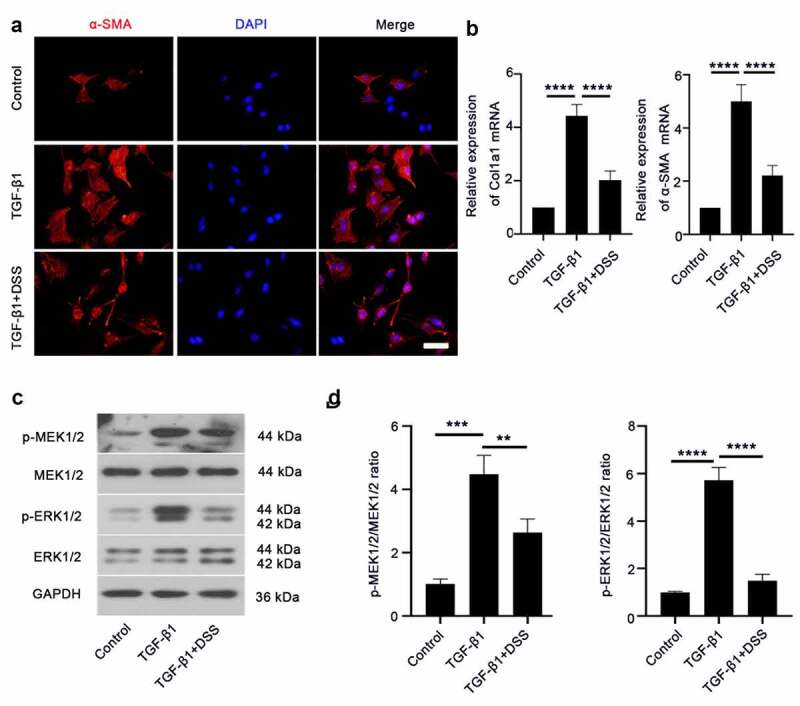
DSS inhibited TGF-β1-induced fibroblast-myofibroblast differentiation via inhibiting the MEK/ERK signaling pathway in NIH3T3 cells. (a) Immunofluorescence was conducted to evaluate the expression of α-SMA (red). Nucleus was stained with DAPI (blue). Scale bar: 50 μm. (b) Relative mRNA expression of Col1a1 and α-SMA. (c, d) Expressions of p-MEK1/2, MEK1/2, p-ERK1/2, ERK1/2 were detected by western blot. GAPDH was conducted as a loading control. One-way ANOVA, **p < 0.01, ***p < 0.001, ****p < 0.0001

COL-I and α-SMA are markers of fibroblast differentiation to myofibroblasts [18]. Hence, to establish if DSS inhibited TGF-β1-induced fibroblast-myofibroblast differentiation, the expression of α-SMA and COL-I was assessed. Immunofluorescence staining result showed that α-SMA expression after DSS treatment was weaker than that in the TGF-β1-treated cells (Figure 2a). The mRNA level of Col1a1 and α-SMA was significantly reduced by DSS administration compared with the TGF-β1-treated cells (Figure 2b). To further explore the mechanism of DSS on inhibiting fibroblast-myofibroblast differentiation, we evaluated the potential role of the MEK/ERK signaling pathway. Western blot assay exhibited that TGF-β1 elevated the expression of MEK1/2 and ERK1/2 phosphorylation. After DSS treatment, the phosphorylation of MEK1/2 and ERK1/2 was decreased (Figure 2c and Figure 2d). These results suggested that DSS might suppress fibroblast-myofibroblast differentiation via inhibiting the MEK/ERK signaling pathway in TGF-β1-induced NIH3T3 cells.

### Effects of DSS on BLM-induced fibrotic lesions in the lung of mice

**Figure 3.**
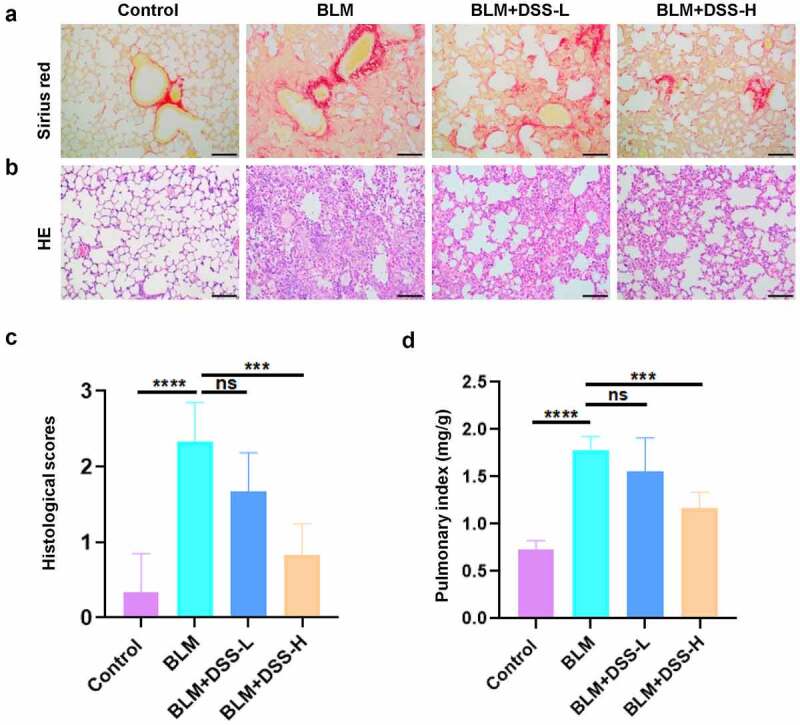
DSS alleviated BLM-induced PF in mice. (a-b) Sections of lung tissue were stained with HE staining and sirius red staining. Scale bar: 100 μm. (c) Histological scores. (d) The pulmonary index was measured. One-way ANOVA, ***p < 0.001, ****p < 0.0001, ns: non-significant

To further examine the effect of DSS *in vivo*, BLM-induced PF model was used. After treated with BLM alone, damaged lung structures, thickened alveolar walls, infiltrated inflammatory cells and accumulated areas of collagen deposition were observed in the lung tissues of mice. However, DSS administration alleviated the results induced by BLM (Figure 3a and Figure 3b). Histological scores analysis indicated that DSS reduced the severity of PF (Figure 3c). Moreover, decreased pulmonary index was found with DSS treatment compared with BLM treatment alone (Figure 3d). Hence, these data exhibited that DSS alleviated BLM-induced fibrotic lesions in the lung of mice.

### Effects of DSS on fibroblast-myofibroblast differentiation in BLM-induced mice

**Figure 4.**
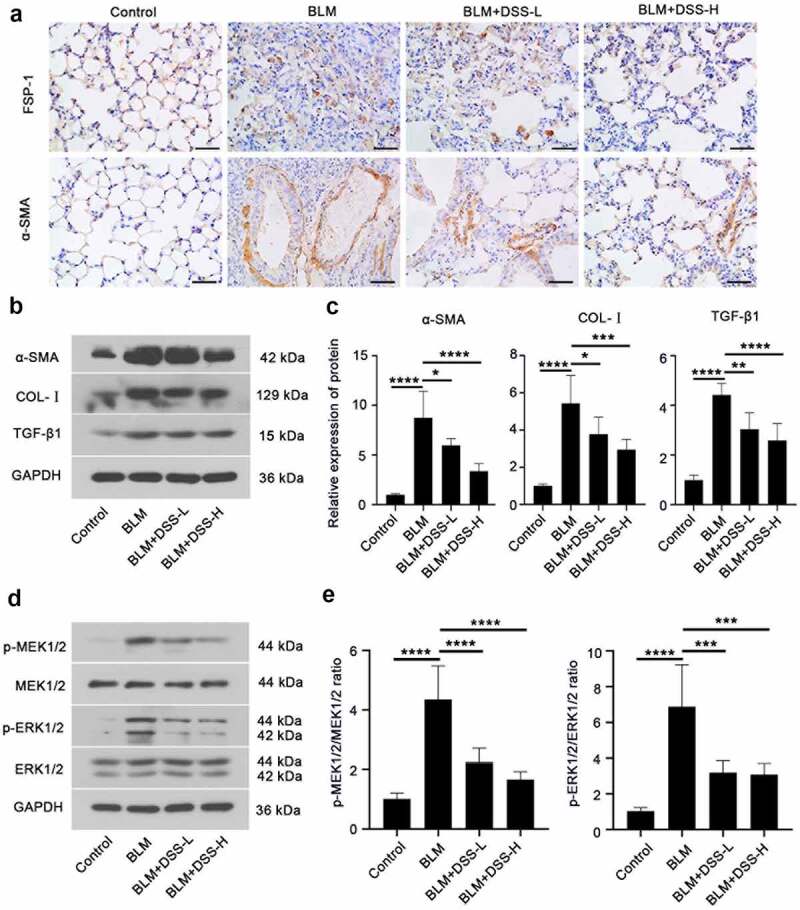
DSS inhibited fibroblast-myofibroblast differentiation via inhibiting the MEK/ERK signaling pathway in BLM-induced mice. (a) Immunohistochemistry analysis of FSP-1 and α-SMA in sections of lung tissues. Scale bar: 50 μm. (b, c) Protein expressions of α-SMA, COL-I, and TGF-β1 were detected by western blot. GAPDH was conducted as a loading control. (d, e) Protein expressions of p-MEK1/2, MEK1/2, p-ERK1/2, ERK1/2 were examined by western blot. GAPDH was conducted as a loading control. One-way ANOVA, *p < 0.05, ***p < 0.001, ****p < 0.0001

To assess whether DSS inhibited fibroblast-myofibroblast differentiation in BLM-induced mice, we investigated the expression of FSP-1, α-SMA and COL-I. FSP-1 – and α-SMA-positive areas were obviously increased in lungs of BLM-treated mice, while DSS (28 and 56 mg/kg) decreased the FSP-1 – and α-SMA-positive areas (Figure 4a). Additionally, western blot results showed that treatment with BLM resulted in an increase in the expression of α-SMA and COL-I in the lung tissues of mice, while DSS reversed the results (Figure 4b and Figure 4c). Meanwhile, decreased protein level of TGF-β1 was observed in DSS-treated mice compared with BLM group (Figure 4b and Figure 4c). Furthermore, we detected the MEK/ERK signaling pathway. In line with *in vitro* results, we found that DSS repressed the phosphorylation of MEK1/2 and ERK1/2 in BLM-treated mice (Figure 4d and Figure 4e). Taken together, these results indicated that DSS inhibited the fibroblast-myofibroblast differentiation via inhibiting the MEK/ERK signaling pathway in BLM-induced mice.

## Discussion

PF is a progressive disease, which can lead to death. With PF onset, fibroblasts will proliferate, migrate and differentiate into myofibroblasts to form fibroblast foci [19]. Although pirfenidone and nintedanib have been used clinically to treat PF, none of these can significantly reduce the mortality of patients [20,21]. In addition, there are few effective drugs that can reverse human PF and prevent chronic development of respiratory failure [22]. Therefore, it is a concern to find new effective and targeted therapies for PF. In this study, we found both *in vitro* and *in vivo* that DSS could inhibit fibroblast proliferation, migration, and differentiation into myofibroblast, likely by repressing the MEK/ERK signaling pathway.

First, we found that DSS significantly improved the cell proliferation caused by TGF-β1 with no effect on cell viability. Meanwhile, we found that DSS inhibited the expression of PCNA, which further confirmed the ability of DSS to inhibit cell proliferation. Consistent with our results, several studies have shown the inhibitory effect of DSS on cell proliferation. For example, Jia et al. has shown that DSS inhibited unusual epidermal proliferation in psoriasis [23]. Zhang et al. has exhibited that DSS can inhibit the proliferation of HSC-T6 in liver fibrosis [24]. Another study has demonstrated that DSS prevents hypoxic pulmonary hypertension in rats by inhibiting the proliferation of pulmonary artery smooth muscle cells in a TGF-β-smad3-associated manner [25]. Interestingly, a research has shown that DSS can promote cell proliferation in Detroit 551 human normal fibroblast cells [26], which does not seem to be consistent with our results. We think it may due to different cells that DSS acts on, and different ways that cells are treated. There may be interactions in this pathological microenvironment, and the regulation mechanism needs to be further explored. Our study showed that DSS inhibited cell proliferation, which implies that DSS has another side in affecting cell proliferation. Therefore, how to reasonably use DSS to achieve the desired therapeutic effect requires more in-depth research in future clinical applications. Then, we found that DSS could inhibit fibroblast-myofibroblast differentiation by reducing the expression of myofibroblast markers α-SMA and COL-I. Additionally, we also found that DSS reduced the expression of the prototypical fibroblast marker FSP-1. Similar with our results, Ji et al. has found that Paeoniflorin inhibited PF stimulated by TGF-β1 through suppressing the expression of FSP-1, COL-I and α-SMA in A549 cells [27]. Vu et al. has shown that interferon-γ prevented IPF via down-regulating the expression of COL-I and α-SMA [18]. Wang et al. has clarified that Fus down-regulated the expression of COL-I and α-SMA in AngII-induced cardiac fibroblasts [28]. Yang et al. has exhibited that INHBA-AS1 silencing alleviated the production of COL-I and α-SMA by targeting miR-141-3p in human hypertrophic scar fibroblasts [29]. Interestingly, several studies have also shown that DSS plays an important role in other diseases such as cardiac fibrosis [30] and liver fibrosis [24]. Moreover, Shao et al. has revealed the effect of two Chinese herbal medicines containing Danshen on anti-pulmonary fibrosis [31]. Lu et al. has shown that DSS suppresses cardiac fibrosis induced by β-adrenergic receptor via negatively regulating ROS-p38 MAPK signaling [16]. Collectively, these results have suggested that DSS has a potential anti-fibrotic effect.

Furthermore, we detected the expression of TGF-β1 in lungs of BLM-induced mice. TGF-β1 is a key driving force of PF [32]. First, TGF-β1 can promote the accumulation of collagen, which is beneficial for organ wound repair. However, too frequent ECM reinforcement with collagen as the main component will turn into progressive fibrosis [33]. Studies in cultured epithelial cells and mesenchymal cells have found that TGF-β1 promotes the production of fibronectin and collagen by regulating the transcriptional activation of related genes [34]. In addition, subcutaneous injection of TGF-β1 is believed to strongly promote collagen accumulation and fibrotic tissue response [35]. Second, TGF-β1 is also involved in inflammation regulation [36,37]. A study has reported that deletion of the TGF-β1 gene in mice will cause the mice to die quickly after birth, which is caused by systemic inflammation [37]. Hence, TGF-β1 is generally considered an inhibitor of excessive inflammation [38]. Regulated inflammation plays an important factor in determining tissue repair, limiting the formation of fibrosis by promoting the decomposition of ECM and inhibiting the accumulation of collagen [33,39,40]. However, TGF-β1 can stimulate fibroblasts to produce ECM and inhibit ECM degradation by matrix metalloproteinases [41]. Last but most important, TGF-β1 can promote the differentiation of fibroblasts into myofibroblasts [42]. A study has shown that the production of myofibroblasts and PF can be observed by transferring the TGF-β1 gene into mouse lung tissue [43]. Zhang et al. has demonstrated that DSS improves pulmonary hypertension by inhibiting TGF-β/Smad3 pathway [25]. In our study, we found DSS treatment (28 and 56 mg/kg) reduced TGF-β1 expression in BLM-induced mice, which further confirmed that DSS could alleviate PF. In line with our study, Liu et al. has indicated that DSS repressed the expression of TGF-β induced by hypoxia on pulmonary hypertension [44]. It is worth noting that we referred to the DSS concentration used in the existing literature [15,45] and then used the body surface area (BSA) normalization method [46] to calculate the concentration of DSS used in our experiments.

Additionally, TGF-β1 and its downstream effector IL-11 have been reported to activate the MEK/ERK signaling pathway to mediate senescence-associated PF [47]. Hence, to further explore the molecular mechanism of DSS on alleviating PF, we detected the expression of the MEK/ERK signaling pathway. The MEK/ERK signaling pathway has been involved in the regulation of pulmonary fibrosis [48]. Moreover, Madala, Satish K et al. have shown that using MEK inhibitor ARRY-142,886 (ARRY) to treat lung fibrosis model mice can prevent lung cell proliferation and increase in total lung collagen, and protect mice from changes in lung function, indicating that MEK/ERK activation plays an important role in human PF [49]. Additionally, recent studies have exhibited the inhibitory effect of DSS on the MEK/ERK signaling pathway in cardiovascular diseases [50,51]. In our study, we found that DSS could reduce the expression of p-MEK1/2 and p-ERK1/2, which indicated that DSS might alleviate PF via inhibiting the MEK/ERK signaling pathway.

## Conclusion

Taken together, our research has proposed that DSS can alleviate TGF-β1 – and BLM-induced PF via inhibiting the conversion of fibroblasts to myofibroblasts, possibly through suppressing the MEK/ERK signaling pathway. Our study provides a new sight for the future PF treatment.

## Data accessibility

All data collected as part of this work are directly presented in the text and figures of the manuscript.

## Highlights

1. DSS alleviated BLM-induced PF in mice. 2. DSS repressed TGF-β1-induced PF in NIH3T3 cells.

3. DSS might be a potential therapeutic drug for PF treatment.
